# Pyomelanin Synthesis in *Alternaria alternata* Inhibits DHN-Melanin Synthesis and Decreases Cell Wall Chitin Content and Thickness

**DOI:** 10.3389/fmicb.2021.691433

**Published:** 2021-08-27

**Authors:** Chantal Fernandes, Marta Mota, Lillian Barros, Maria Inês Dias, Isabel C. F. R. Ferreira, Ana P. Piedade, Arturo Casadevall, Teresa Gonçalves

**Affiliations:** ^1^CNC—Center for Neuroscience and Cell Biology of Coimbra, Coimbra, Portugal; ^2^FMUC—Faculty of Medicine, University of Coimbra, Coimbra, Portugal; ^3^Mountain Research Center (CIMO), Polytechnic Institute of Bragança, Bragança, Portugal; ^4^Laboratory of Separation and Reaction Engineering - Laboratory of Catalysis and Materials (LSRE-LCM), Polytechnic Institute of Bragança, Bragança, Portugal; ^5^Centre for Mechanical Engineering, Materials and Processes, Department of Mechanical Engineering, University of Coimbra, Coimbra, Portugal; ^6^Department of Molecular Microbiology and Immunology, Johns Hopkins Bloomberg School of Public Health, Baltimore, MD, United States

**Keywords:** pyomelanin, DHN-melanin, *Alternaria alternata*, chitin, L-tyrosine, L-phenylalanine, melanin

## Abstract

The genus *Alternaria* includes several of fungi that are darkly pigmented by DHN-melanin. These are pathogenic to plants but are also associated with human respiratory allergic diseases and with serious infections in immunocompromised individuals. The present work focuses on the alterations of the composition and structure of the hyphal cell wall of *Alternaria alternata* occuring under the catabolism of L-tyrosine and L-phenylalanine when cultured in minimal salt medium (MM). Under these growing conditions, we observed the released of a brown pigment into the culture medium. FTIR analysis demonstrates that the produced pigment is chemically identical to the pigment released when the fungus is grown in MM with homogentisate acid (HGA), the intermediate of pyomelanin, confirming that this pigment is pyomelanin. In contrast to other fungi that also synthesize pyomelanin under tyrosine metabolism, *A. alternata* inhibits DHN-melanin cell wall accumulation when pyomelanin is produced, and this is associated with reduced chitin cell wall content. When *A. alternata* is grown in MM containing L-phenylalanine, a L-tyrosine percursor, pyomelanin is synthesized but only at trace concentrations and *A. alternata* mycelia display an albino-like phenotype since DHN-melanin accumulation is inhibited. CmrA, the transcription regulator for the genes coding for the DHN-melanin pathway, is involved in the down-regulation of DHN-melanin synthesis when pyomelanin is being synthetized, since the *CMRA* gene and genes of the enzymes involved in DHN-melanin synthesis pathway showed a decreased expression. Other amino acids do not trigger pyomelanin synthesis and DHN-melanin accumulation in the cell wall is not affected. Transmission and scanning electron microscopy show that the cell wall structure and surface decorations are altered in L-tyrosine- and L-phenylalanine-grown fungi, depending on the pigment produced. In summary, growth in presence of L-tyrosine and L-phenylalanine leads to pigmentation and cell wall changes, which could be relevant to infection conditions where these amino acids are expected to be available.

## Introduction

*Alternaria alternata* is the most common species of the genus *Alternaria* and it is the major environmental allergen associated with asthma and allergic rhinitis (Knutsen et al., 2012; Denning et al., 2014; Fukutomi and Taniguchi, 2015), also described as an opportunistic agent of infection in immunocompromised patients. *Alternaria* spp. are dematiaceous fungi characterized by the accumulation of melanin in the cell wall of conidia and hyphae. Melanins are a group of related pigments that share common physical and chemical characteristics (Eisenman and Casadevall, 2012). They are formed by the oxidative polymerization of phenolic or indolic compounds. In fungi, melanins may be synthesized from endogenous substrates via a 1,8-dihydroxynaphthalene (DHN) intermediate or alternatively, from L-3,4-dihydroxyphenylalanine (L-DOPA) (Eisenman and Casadevall, 2012). Other melanins called alkaptomelanin are derived from L-tyrosine (Yabuuchi and Ohyama, 1972). This pigment is referred as alkaptomelanin when is produced by humans with alkaptonuria, a rare disease due to mutations in the homogentisate dioxygenase gene but is usually called pyomelanin when synthesized by microbes. Pyomelanin is a water-soluble brown pigment first identified in *Pseudomonas aeruginosa* (Yabuuchi and Ohyama, 1972).

*A. alternata* accumulates DHN-melanin in hyphal and conidial cell walls (Carzaniga et al., 2002). This pigment is synthesized by the 1,8-dihydroxynaphthalene (DHN) pathway with 1,8-DHN as intermediate (Kimura and Tsuge, 1993; Tseng et al., 2011; Eisenman and Casadevall, 2012). Three classes of melanin mutants have been reported in *A. alternata*: two albino mutants (Δ*cmra*, Δ*alm*), a lightbrown (Δ*brm1*) and a brown mutant (Δ*brm2*) (Kimura and Tsuge, 1993; Fetzner et al., 2014). The color phenotype of these mutants depends on the gene deleted, since the pathway intermediates lead to the shunt products like flaviolin and/or 2-hydroxyjuglone, that are colored (Lee et al., 2003). The melanin-deficient strains are more sensitive to UV light (Kawamura et al., 1999). The transcription factor CmrA, the regulator for this melanin route was first discovered in *Colletotrichum lagenarium* and *Magnaporthe grisea* in a screen for insertional mutants with lowered pathogenicity (Tsuji et al., 2000) and was then characterized in *Alternaria brassicicola* and *A. alternata*, where it controls the expression of at least three structural genes for melanin biosynthesis (Cho et al., 2012; Fetzner et al., 2014).

**FIGURE 1:**
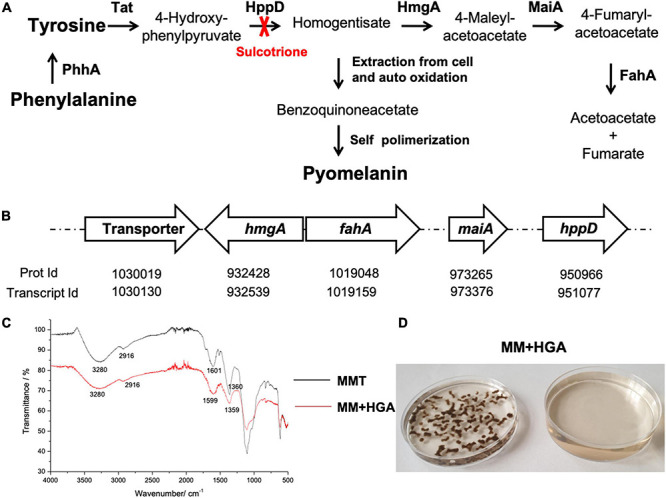
Pyomelanin synthesis in *A. alternata* by L-Tyrosine degradation to HGA. **(A)** L-Tyrosine degradation pathway and pyomelanin synthetic pathway. The enzymes indicated in bold as follows: PhhA, phenylalanine hydroxylase; Tat, L-tyrosine aminotransferase; HppD, 4-hydroxyphenylpyruvate dioxygenase; HmgA, homogentisate dioxygenase; MaiA, maleylacetoacetate isomerase; FahA, fumarylacetoacetate hydrolase. **(B)** Arrangement of the genes putatively involved in these pathways in *A. alternata*. The pyomelanin pigment derives from L-tyrosine through formation of 4-hydroxyphenylpyruvate and HGA which conversion is inhibited by sulcotrione. HGA is catabolized into acetoacetate and fumarate and depending on the HmgA activity, HGA may accumulates and can be secreted from the cell (Yabuuchi and Ohyama, 1972). Then, after auto-oxidation into benzoquinoneacetate and self-polymerization, forms a brown pigment, pyomelanin (Rodríguez et al., 2000). The genes encoding the enzymes involved in these pathways were found in *A. alternata* SRC1lrK2f v1.0 genome, at JGI: http://genome.jgi.doe.gov (The Genome Portal of the Department of Energy Joint Genome Institute). The transporter (Prot Id 985975) may be involved in the transport of HGA out of the cell. **(C)** FTIR spectra of the pigments obtained from culture medium of *A. alternata* grown in MMT (black) and in MM + HGA (red). **(D)** Culture medium and mycelia of *A. alternata* grown in MM + 5.5 mM HGA.

Pyomelanin biosynthesis occurs by oxidative polymerization of homogentisate (HGA), an intermediate of the catabolism of L-tyrosine or L-phenylalanine (Lehninger, 1975; Figure 1A). The degradation of L-tyrosine to acetoacetate and fumarate requires the activity of the enzymes 4-hydroxyphenylpyruvic acid dioxygenase (4-HPPD) and homogentisic acid oxidase (HGA-oxidase). When HGA production exceeds HGA-oxidase activity, HGA is excreted from the cell (Yabuuchi and Ohyama, 1972; Ruzafa et al., 1994; Kotob et al., 1995). Auto-oxidation followed by self-polymerization of HGA leads to pyomelanin. Additionally, deletion of the gene that encodes for 4-HPPD or inhibition of this enzyme by specific inhibitor such as sulcotrione, impair the synthesis of pyomelanin (Ruzafa et al., 1995; Turick et al., 2010). This water-soluble pigment binds to the surface of hyphae (Heinekamp et al., 2013), and protects the fungus against reactive oxygen species (ROS) (Schmaler-Ripcke et al., 2009) and cell wall stress (Valiante et al., 2009). This pigment was associated with virulence in both *Pseudomonas aeruginosa* and *Cryptococcus neoformans*, since pyomelanin reduced the phagocytosis rate (Frases et al., 2007; Rodríguez-Rojas et al., 2009). However, in *Aspergillus fumigatus*, the mutants deficient for pyomelanin biosynthesis do not differ in virulence when tested in a murine infection model for invasive pulmonary aspergillosis Keller et al., 2011; Heinekamp et al., 2013), even though the transcription of the L-tyrosine degradation gene cluster is increased under cell wall stress (Jain et al., 2011) and when exposed to immature dendritic cells or human neutrophils (Morton et al., 2011).

*Alternaria* spp. secrete a wide variety of proteases during spore germination and growth (Boitano et al., 2011; Snelgrove et al., 2014) that can potentially degrade host proteins. Consequently, the amino acids L-tyrosine and L-phenylalanine are likely to be available to fungal cells during human and animal infection. Furthermore, normal human plasma L-tyrosine levels range from 21 to 107 μM (Gressner, 2007; Schmaler-Ripcke et al., 2009). The higher concentrations are located in neural tissues as L-tyrosine readily crosses the blood brain barrier can be used as a precursor of the neurotransmitters norepinephrine and dopamine (Fernstrom and Fernstrom, 2007). With regards to L-phenylalanine levels, reference blood phenylalanine concentrations are between 21 and 137 μmol/L in healthy children up to the age of 18 years and in adults, 35–85 μmol/L (Cleary et al., 2013). However, individuals with phenylketonuria, a disorder caused by an impaired conversion of L-phenylalanine to L-tyrosine (due to a deficiency in activity of a renal enzyme L-phenylalanine hydroxylase), may have in an increased in plasma L-phenylalanine concentrations, with values above 1.0 mM (Mitchell, 2013).

In this context, it is likely that L-tyrosine and L-phenylalanine are available during infection or during human lung colonization, leading to the transcription of the L-tyrosine degradation genes from *A. alternata* and catabolism of these amino acids. In the present work, we describe the production of pyomelanin in *A. alternata* during L-tyrosine catabolism and report the consequent inhibition of DHN-melanin synthesis together with changes in chitin content and cell wall structure.

## Materials and Methods

### Organisms and Media

*A. alternata* was kindly provided by R. M. B. Ferreira (Instituto Superior de Agronomia, Universidade de Lisboa, Lisbon, Portugal) and was stored at –80°C. Cultures were grown on PDA (Potato Dextrose Agar; Difco), minimal medium [MM; 1 L contained 20 mL of salts solution (NaNO_3_ 300 g/L, KCl 26 g/L, MgSO_4_.7H_2_O 24.65 g/L), 9 g of glucose, 12 mL of phosphate buffer 1 M pH 6.8 (KH_2_PO_4_ 68 g/L, K_2_HPO_4_ 87.1 g/L), 1 mL of Clive Roberts trace elements solution (FeSO_4_.7H_2_O 1 g/L, ZnSO_4_.7H_2_O 8.8 g/L, CuSO_4_.5H_2_O 400 mg/L, MnSO_4_.H_2_O 150 mg/L, Na_2_B_4_O_7_.10H_2_O 100 mg/L, (NH_4_)_6_Mo_7_O_24_.4H_2_O 50 mg/L and 15 g/L of agar for solid media^1^)]. MM liquid medium was sterilized by filtration and solid medium by autoclaving at 121°C for 20 min. For pyomelanin production, the MM medium was added with 5.5 mM of L-tyrosine (Sigma, T-8566) (referred as MMT) or 5.5 mM of L-phenylalanine (Sigma, P-5482) (referred as MMP) and heated under agitation until solubilized and then sterilized by filtration. Solutions with 5.5 mM L-valine (Sigma, V-0513) and 5.5 mM L-glutamine (Sigma, G-3126) were also used as control in MM.

### Preparation of Inocula and Growth Conditions

The procedures used to prepare inocula were as previously described (Anjos et al., 2012). Briefly, spores of *A. alternata* were collected from 2-weeks-old plate cultures grown at 28°C with alternating 16-h light 8-h dark cycle under a BLB blacklight blue lamp (15 W). The mycelium was submerged in liquid MM and scraped with an inoculation loop, and this suspension was used as the inoculum. Liquid *A. alternata* cultures were cultivated on MM at 28°C with constant orbital shaking at 130 rpm with alternating 16-h light 8-h dark cycle (Fernandes et al., 2014).

### *A. alternata* Growth Curve, Quantification of the Pigment Released and Accumulated From Cultures

Several Erlenmeyer flasks with 50 mL of MM, MM supplemented with 5.5 mM of L-tyrosine (MMT) or MM supplemented with 5.5 mM of L-phenylalanine (MMP) were inoculated with 1 × 10^6^ *A. alternata* conidia and incubated on a rotary shaker at 120 rpm at 28°C under 16 h-alternating light enriched with UV and 8 h dark. Every 24 h, for 10 days, Erlenmeyer flasks content were analyzed for pigment released and fungal growth determined by dry weight. The mycelial mats were harvested by filtration in a steel filter. The pigment was released and analyzed by absorbance measurements at 405 nm in a Spectra Max Plus384 spectrophotometer (Molecular Devices, LLC) (Schmaler-Ripcke et al., 2009). To estimate fungal biomass growth, the material was freeze-dried and weighed. The pigment bound to the fungal cell wall was quantified and expressed per mg of dry weight. For quantification, the samples were suspended in distilled H_2_O, sonicated (Sonics and Materials, Inc.) and extracted with NaOH 1 M as described before (Fernandes et al., 2015) and pigments were quantified spectrophotometrically at 405 nm.

### Effect of Sulcotrione, Pyroquilon, and Glyphosate on Pigment Release From *A. alternata*

To evaluate the effect of sulcotrione, pyroquilon and glyphosate on pigment synthesis by *A. alternata*, we inoculated MM, MMT and MMP liquid medium with, and without, 100 μg/mL of sulcotrione, pyroquilon and glyphosate. The pigments released in the supernatant were quantified at 405 nm after 3 days of incubation and the mycelium was harvested.

Additionally, to understand whether *A. alternata* DHN-melanin pathway is activated under L-tyrosine metabolism, we analyzed the supernatants of *A. alternata* grown in MM and MMT, with and without 100 μg/mL pyroquilon, to follow the production of flaviolin, a shunt product released into the medium when DHN-melanin pathway is inhibited by pyroquilon. The supernatants of *A. alternata* grown in MM and MMT, and grown under these conditions with pyroquilon were filtered through a 0.2 μm sterile syringe cellulose acetate filter (VWR, United States) and concentrated to 100 mg/mL for chromatographic analysis performed in a Dionex Ultimate 3000 UPLC (Thermo Scientific, San Jose, CA, United States) system equipped with a diode array detector coupled to a electrospray ionization mass detector (LC-DAD-ESI/MSn), a quaternary pump, an auto-sampler (kept at 5°C), a degasser and an automated thermostatic column compartment.

Chromatographic separation was achieved with a Waters Spherisorb S3 ODS-2 C18 (3 μm, 4.6 mm × 150 mm, Waters, Milford, MA, United States) column thermostatic at 35°C.

The solvents used were: (A) 0.1% formic acid in water, (B) acetonitrile. The elution gradient established was isocratic 15% B (5 min), 15 B–20% B (5 min), 20–25% B (10 min), 25–35% B (10 min), 35–50% B (10 min), and re-equilibration of the column, using a flow rate of 0.5 mL/min. Double online detection was carried out in the DAD using 280, 370, 480, and 520 nm as preferred wavelengths and in a mass spectrometer (MS) connected to HPLC system via the DAD cell outlet.

MS detection was performed in negative mode, using a Linear Ion Trap LTQ XL mass spectrometer (Thermo Finnigan, San Jose, CA, United States) equipped with an ESI source. Nitrogen served as the sheath gas (50 psi); the system was operated with a spray voltage of 5 kV, a source temperature of 325°C, a capillary voltage of –20 V. The tube lens offset was kept at a voltage of –66 V. The full scan covered the mass range from *m*/*z* 100 to 1,500. The collision energy used was 35 (arbitrary units). Data acquisition was carried out with Xcalibur^®^ data system (Thermo Finnigan, San Jose, CA, United States).

### Characterization of the Pigments Released by Ultraviolet-Visible and Fourier Transform Infrared Spectroscopy (FTIR)

A volume of 200 mL of MM with 5.5 mM of L-tyrosine or 5.5 mM HGA (D1050, TCI) was inoculated with 5 × 10^6^ conidia of *A. alternata* and incubated for 4 days on a rotary shaker at 120 rpm at 28°C under 16 h-alternating light enriched with UV and 8 h dark. The mycelial mats were harvested by filtration in a steel filter and the supernatant medium collected. The supernatant was filtered through a 0.2 μm cellulose acetate filter membrane, dialyzed overnight at room temperature against distilled H_2_O and lyophilized.

The infrared spectra of the pigments were recorded on a ThermoNicolet IR300 Fourier transform infrared spectrometer, equipped with a deuterated triglycine sulfate (DTGS) detector and a KBr beam splitter. Thermo Scientific Nicolet Smart Orbit diamond ATR accessory was also used with a resolution of 1 cm^–1^.

### Quantification of Expression of the Transcription Factor *CMRA* and of the Genes *PKSA*, *BRM1*, and *BRM2* of the DHN-Melanin Synthesis

Total RNA isolation was performed with the Bioline ISOLATE II RNA Mini Kit II BIO-52072 (Meridian Bioscience) following manufacturer’s instructions. Reverse transcription (RT) of 3 μg of total RNA was performed with the 1st Strand cDNA synthesis kit for RT-PCR (Roche) according to the manufacturer’s instructions. The relative quantification of the expression of *CMRA* and of the polyketide synthase gene (*PKSA*), scytalone dehydratase encoding gene (*BRM1*) and 1,3,8-trihydroxynaphthalene (THN) reductase gene (*BRM2*) was performed with the ß-tubulin rRNA gene as the reference. Real-time RT-quantitative PCRs (RT-qPCRs) were performed in a LightCycler 2.0 (Roche Diagnostics) with a EvaGreen supermix (BioRad). The primers used for gene quantification were based on Fetzner et al., 2014: CmrF: GAAATGTCACCTGCGCAAAC; CmrR: GTCTTGGGCTGCGATAATG; PksF: GATTGCCATC GTCGGTATG; PksR: GGCTCATCGATGAAGCAAC, Brm1F: CTACGAGTGGGCAGACAG; Brm1R: GTACCGCCGATGAA GTGCTG; Brm2F: CCGTGGTATCGGAAAGGC; Brm2R: GA AGTGGGCAACAACGTCAT; BtubF: GTTGAGAACTCAGAC GAGAC; BtubR: CATGTTGACGGCCAACTTC. The expression level of each gene was normalized to the value of the reference (ß-tubulin rRNA gene) according to the 2^–ΔΔCT^ method (Livak and Schmittgen, 2001).

### Quantification of Chitin and β-Glucan

*A. alternata* was grown in liquid MM, MMT, and MMP with or without 100 μg/mL pyroquilon at 28°C, at 120 rpm for 3 days under alternating 16-h light 8-h dark cycle. Quantification of cell wall chitin content was based on the measurement of the glucosamine released by acid hydrolysis of purified cell walls (Fernandes et al., 2014). Cells were disrupted by sonication and washed five times with 1 M NaCl and extracted in SDS-MerOH buffer [50 mM Tris, 2% sodium dodecyl sulphate (SDS), 0.3 M ß-mercaptoethanol, 1 mM EDTA; pH 8.0] at 100°C for 10 min, then washed in dH_2_O. Cell wall pellets were resuspended in sterile dH_2_O, freeze dried, and the dry weight of recovered cell walls was measured (Munro et al., 2007). Chitin contents were determined by measuring the glucosamine released by acid hydrolysis of purified cell walls (Kapteyn et al., 2000).

Quantification of glucan contents was performed with the aniline blue assay. The mycelium was washed and then sonicated in 1 M NaOH, and incubated at 52°C for 30 min. β-1,3-glucan concentration was determined by aniline blue fluorescence (Kahn et al., 2006).

### Transmission Electron Microscopy (TEM)

The hyphal cell wall structure was evaluated by TEM. Liquid *A. alternata* cultures were cultivated on MM, MMT, MMP and on these media added with 75 μg/mL of pyroquilon, at 28°C with constant orbital shaking at 180 rpm for 3 days with the day-night cycles described above.

To evaluate the amount and the distribution of melanin accumulated in the cell wall of *A. alternata* grown in MM, MMT, MMP, we obtained melanin ghosts by chemical and enzymatic removal of the other cellular structures as previously described (Dadachova et al., 2007; Fernandes et al., 2015).

The samples were fixed with 2.5% glutaraldehyde in 0.1 M sodium cacodylate buffer (pH 7.2) for 2 h. Post fixation was performed using 1% osmium tetroxide for 1 h. After they were rinsed with the buffer, the samples were dehydrated in a graded ethanol series (30–100%), impregnated, and embedded in Epoxy resin (Fluka Analytical). Ultrathin sections (80 nm) were mounted on copper grids (300 mesh). Observations were carried out on an FEI-Tecnai G2 Spirit Bio Twin transmission electron microscope at 100 kV.

### Scanning Electron Microscopy (SEM)

*A. alternata* was grown as described for TEM, washed twice in PBS and the mycelia were placed over silicon (Si) substrates and then chemically fixed with 5% glutaraldehyde solution for 15 min. The dehydration process, as previously described (Carvalho et al., 2016), consisted in immersing the set mycelia in solution with increasing concentrations of ethanol in the ethanol:water ratio: 0:100, 20:80, 50:50, and 100:0. In each solution the samples were kept at room temperature for 15 min. After the final step the ethanol was aspirated and the alcohol residue removed by evaporation overnight. Before SEM observation the Si/cells were sputter coated with a 10 nm thick gold layer, to turn the biological material electron conductive. The observations were made using a Philips X30 equipment.

### Statistical Analysis

Analysis of variance was performed by One-way ANOVA followed by Tukey multiple comparison test, using Prism (version 5) software (GraphPad Software, Inc., La Jolla, CA). Mann-Whitney test was used for qPCR quantification (Prism). Data are presented as the means ± standard errors of the means (SEMs) and differences were considered significant at *P*-values of < 0.05.

## Results

### Production of Melanin Pigments in *A. alternata*

**FIGURE 2:**
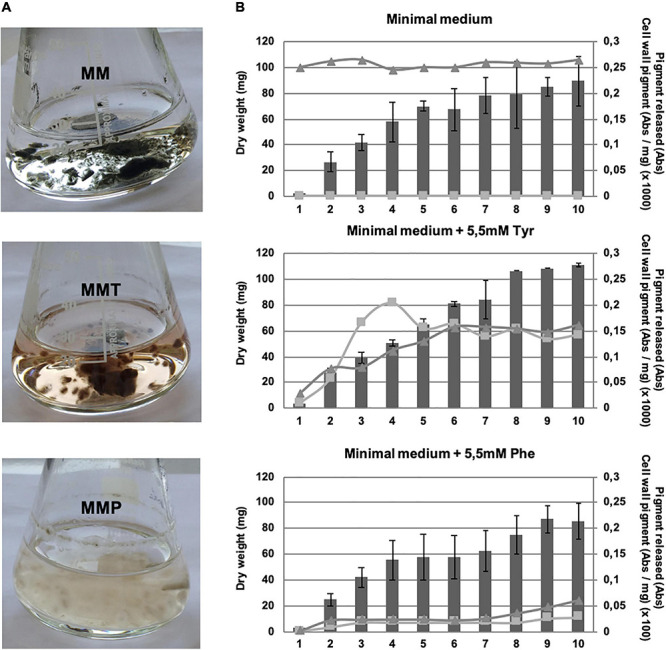
*A. alternata* melanin synthesis during mycelial growth. **(A)** Pigment formation of *A. alternata* grown in MM, in MM supplemented with 5.5 mM of L-tyrosine (MMT) and in MM supplemented with 5.5 mM of L-phenylalanine (MMP) after 3 days of growth. **(B)** Mycelial growth and quantification of the melanin content in *A. alternata* cells and released in liquid cultures over 10 days. Bars, mycelium dry weight; squares, pigment released into the medium, given as the absorbance at 405 nm (Abs); triangles, cell wall pigment content, measured by determination of the absorbance at 405 nm, as described in section “Materials and Methods”, and given as the absorbance (Abs) per milligram (dry weight) of mycelia (×1,000 for the scale of MM and MMT growth and × 100 for the MMP growth scale).

When grown in MM, *A. alternata* synthetized a black pigment that was not released into the medium. In MM supplemented with 5.5 mM of L-tyrosine (MMT), *A. alternata* produced a brown pigment bound to the hypha that was also released into the medium (Figure 2A). However, in MM supplemented with 5.5 mM of L-phenylalanine (MMP), a L-tyrosine precursor, *A. alternata* mycelium was pale and released a low amount of pigment into the medium (Figure 2A). As a control of the effect of amino acid catabolism on the melanisation, we used L-valine and L-glutamine in the same conditions as L-tyrosine and L-phenylalanine, but we did not observe any difference in relation to the control (MM) (results not shown). We also grew *A. alternata* in MM supplemented with phenylacetate to evaluate if the phenyl group might inhibit the DHN-melanin synthesis. In the presence of 0.2 and 0.02 g/L of phenylacetate, *A. alternata* mycelium mat was as black as with MM.

We also quantified the growth and the pigment accumulated in the cell wall hyphae and released into the medium along 10 days (Figure 2B). The growth was not enhanced or inhibited by the presence of L-tyrosine or L-phenylalanine. In MM, the mycelium was black from the very beginning of germination and in both MMT and MMP, the melanisation increased over time. Curiously, with MMT the pigment released by *A. alternata* reached a peak at day 4 and then decreased; this is possibly due to binding to the hyphal cell wall (Figure 2B, middle panel).

### *A. alternata* Pigment Identification

**FIGURE 3:**
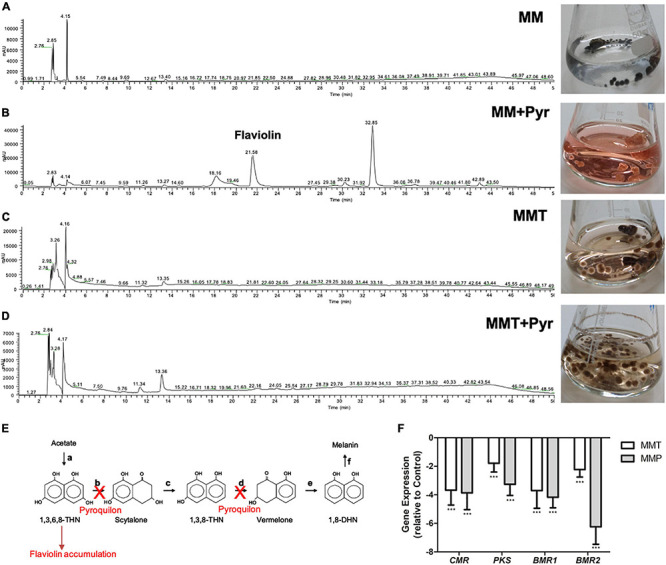
DHN-melanin pathway inhibition in *A. alternata* under tyrosine metabolism. **(A–D)** Chromatographic separation of the components of the supernatants of *A. alternata* grown in **(A)** MM, **(B)** MM + pyroquilon, **(C)** MMT and **(D)** MMT + pyroquilon and respective growth in the Erlenmeyers. In MM + pyroquilon, the DHN-melanin pathway is inhibition by pyroquilon leads to the released of the carmine shunt product flaviolin. **(E)** Biosynthetic pathway leading to the formation of DHN-melanin from acetate and identification of the enzymes involved [**(a)**, polyketide synthase; **(b)**, T4HN reductase; **(c)**, scytalone dehydratase; **(d)**, T3HN reductase; **(e)**, vermelone dehydratase; **(f)**, several candidate enzymes for this step] in fungi. 1,3,6,8-THN = 1,3,6,8-tetrahydroxynaphthalene; 1,3,8-THN = 1,3,8-trihydroxynaphthalene; 1,8-DHN = 1,8-dihydroxynaphthalene. Pyroquilon inhibits the T4HN reductase and the T3HN reductase which leads to the accumulation of flaviolin (Wheeler and Klich, 1995). **(F)** Analysis of the expression of *CMRA*, *PKS*, *BRM1*, *BRM2* in *A. alternata* mycelium cultured in MM, MMT and MMP. Strains were grown for 3 days before mycelium was harvested and RNA isolated. RT-PCR was performed as described in section “Materials and Methods” with the ß-tubulin gene as standard and in comparison to the control MM. Results are the mean ± standard errors of the means (SEMs) of triplicates of three independent experiments (Mann-Whitney, Prism): ***P < 0.001.

Since *A. alternata* is known to synthesize DHN-melanin (Kimura and Tsuge, 1993), we grew *A. alternata* in MM with 50 μg/mL of pyroquilon, a DHN-melanin synthesis inhibitor. Under this condition, *A. alternata* mycelial mat changed from black to a reddish color and released a typically reddish pigment, a shunt product of the DHN-melanin inhibition (Figures 3A–D). When *A. alternata* was grown in MMT or in MMT + pyroquilon and in MMP or in MMP + pyroquilon this reddish pigment was not observed. To determine if the presence of pyomelanin masked the release of the reddish pigment, under growth in MMT or in MMT + pyroquilon we performed a chromatographic analysis of the supernatant. Only the culture medium resulting from the growth in MM + pyroquilon led to the detectable shunt intermediate flaviolin (Figures 3A–D).

To understand the reason of DHN-melanin lack of accumulation in *A. alternata* cell wall under the metabolism of L-tyrosine and L-phenylalanine, we determined the level of gene expression of the *CMR* gene, the transcription factor of the genes coding for the melanin pathway, and of the genes encoding the proteins involved in DHN-melanin synthesis (Figure 3E). We observed a statistical significative decrease in the expression of all these genes in *A. alternata* grown in MMT and MMP in relation to growth in MM (Figure 3F).

The pathway for pyomelanin synthesis from L-tyrosine catabolism was previously elucidated (Figure 1A; Boylan et al., 1987; Schmaler-Ripcke et al., 2009; Rodríguez-Rojas et al., 2009). The open reading frames encoding the enzymes putatively involved in L-tyrosine degradation and in the HGA formation were identified in *A. alternata* genome by a BLAST search at the JGI database^2^ (Figure 1B). The catabolism of L-tyrosine begins with a α-ketoglutarate dependent transamination through the L-tyrosine transaminase to *para*-hydroxyphenylpyruvate. Then, HppD (Protein Id: 950966) catalyzes the formation of HGA that yield to 4-maleylacetoacetate by the action of a HmgA homogentisate dioxygenase (Protein Id: 932428) and then to 4-fumarylacetoacetate and acetoacetate + fumarate in two reactions catalyzed by the maleylacetoacetate isomerase (Protein Id: 973265) and the fumarylacetoacetate hydrolase (Protein Id: 1019048), respectively. Depending on the activity of these enzymes, HGA may accumulate on the cell and can be excreted and auto oxidizes to benzoquinoneacetate that self-polymerizes into pyomelanin. Interestingly, an open reading frame encoding a putative transporter (Protein Id: 1030019) is present in this operon. The protein encoded by this gene may be involved in the transportation of HGA out of the cell (Figure 1B). The presence of these genes in *A. alternata* genome confirmed that *A. alternata* is provided with the enzymes needed for the synthesis of pyomelanin upon the L-tyrosine catabolism (Figures 1A,B). The pyomelanin synthesis inhibitor sulcotrione (100 μg/mL) reduced pigment released by *A. alternata* grown in MMT and prevented formation of pigment when cultivated in MMP. FTIR spectroscopy was used to analyze the pigment released by *A. alternata* grown in MMT and in MM + HGA (Figure 1C). The spectra are equivalent, confirming the similarity of the pigments produced under these two different conditions. The spectra exhibit a broad absorption at 3,280 cm^–1^, due to OH groups, the stretching vibrations for aliphatic CH bonding appear at 2,916 cm^–1^ and the carboxylate stretching vibrations (COO^–^) are detectable at 1,600 (1,601 for MMT and 1,599 for MM + HGA) and 1,360 for MMT (1,359 for MM + HGA) cm^–1^. It is established that pyomelanin pigment results from the polymerization of HGA (Rodríguez et al., 2000). Since we obtained a high level of identity between the FTIR spectra of the pigments synthesized from *A. alternata* cultured with tyrosine or HGA, this indicates that *A. alternata* produces pyomelanin when cultivated with L-tyrosine. As we can see in Figure 1D, the mycelia and the culture medium are brown as when *A. alternata* is grown in MMT (Figure 3C). The amount of melanin extracted from the medium of *A. alternata* grown in MMP was too low for FTIR spectral analysis.

### Ultrastructural Changes in *A. alternata* Hypha Grown in MM, MMT, and MMP Medium

**FIGURE 4:**
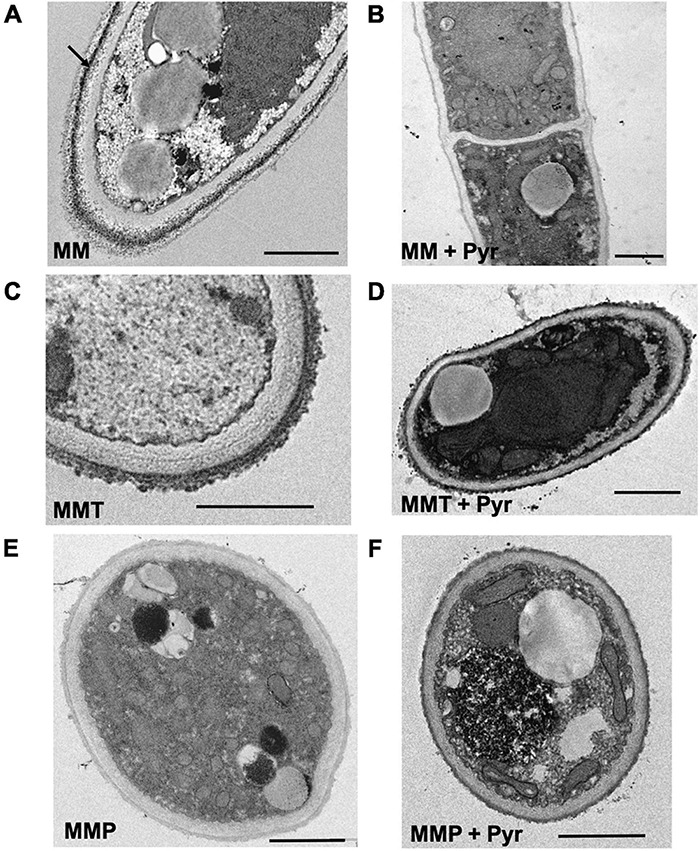
Transmission electron microscopic observations of the hyphae and hyphal cell wall morphology of *A. alternata* grown under different conditions: **(A)** MM, **(B)** MM with the presence of 50 μg/mL of pyroquilon, **(C)** MMT, **(D)** MMT supplemented with 50 μg/mL of pyroquilon, **(E)** MMP and **(F)** MMP + 50 μg/mL of pyroquilon, after 3 days of growth. Arrow in **(A)** show an internal melanin layer. Bars, 1,000 nm.

Transmission electron microscopy (TEM) and scanning electron microscopy (SEM) were used to appraise the ultrastructural changes in *A. alternata* hyphae grown in MM, MMT, or MMP medium and under these conditions plus pyroquilon. Using TEM, we observed that when grown in MM medium, under DHN-melanin synthesis conditions, *A. alternata* cell wall showed an internal electron-dense material layer (Figure 4, arrow), consistent with DHN-melanin being imbedded in the cell wall. When grown in MM + pyroquilon, this internal electron-dense material vanished (Figure 4). Yet, when *A. alternata* was grown in MMT (with or without pyroquilon), the electron-dense material is not internally distributed but is located at the cell wall surface (Figure 4). We also observed that the cell wall surface is rougher due to the presence of a superficial large electron-dense layer of pyomelanin (Figure 4). This electron-dense layer, supposedly of pyomelanin, is also present to a lower extent in *A. alternata* grown in MMP both in the presence of absence of pyroquilon (Figure 4).

**FIGURE 5:**
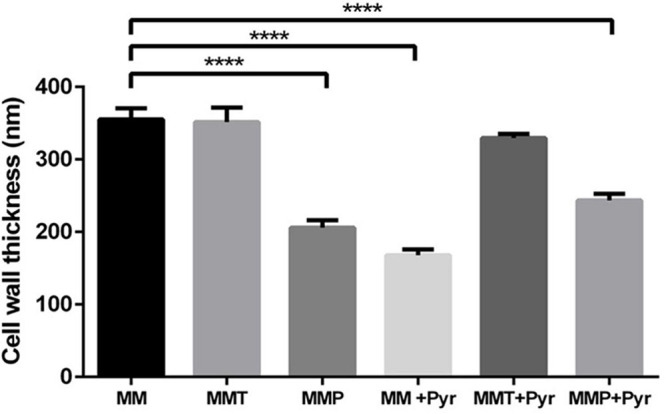
Measurement of the *A. alternata* cell wall when grown in MM, MMT, MMP and under these conditions with pyroquilon. *A. alternata* was cultivated in liquid cultures for 3 days with alternating 16-h light and 8-h dark periods. Results are the mean ± the standard error of the mean of triplicates of three independent experiments (Student’s *t*-test). ****P < 0.0001.

From TEM images we quantified the cell wall thickness and observed differences in the cell wall thickness depending on the growth media (Figure 5). We observed that the conditions leading to higher pigment cell wall contents (MM and MMT) presented significantly thicker cell walls than when *A. alternata* was grown in MMP or MM supplemented with pyroquilon (Figure 5). When *A. alternata* was grown in MMT or MMP, and under these conditions plus pyroquilon, there was no significant reduction in cell wall thickness, indicating the absence of DHN-melanin from the cell walls does not lead to increased cell wall thickness. Otherwise, when MM or MMP were supplemented with pyroquilon the cell wall decreased in relation to control (MM). Also, MMP supplementation with pyroquilon does not change the cell wall thickness when compared with MMP alone (Figure 5).

**FIGURE 6:**
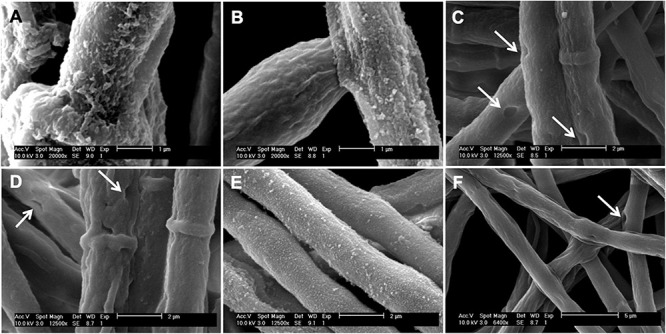
Scanning electron micrographs of the *A. alternata* hyphae grown in **(A)** MM, **(B)** MMT, **(C)** MMP, **(D)** MM + pyroquilon, **(E)** MMT + pyroquilon and **(F)** MMP + pyroquilon. Arrows indicate holes in the cell wall when the fungus is grown in MM + pyroquilon, MMP and MMP + pyroquilon.

By SEM, we observed decorations coating *A. alternata* grown in MM (Figure 6A) and in MMT and MMT + pyroquilon (Figures 6B,E). These decorations were not present when *A. alternata* was grown in MM + pyroquilon (Figure 6D), MMP or MMP + pyroquilon (Figures 6C,F), indicating that these decorations are due to DHN-melanin and the surface layer (the most external observed when grown in MM; Figure 4A) and to a dense pyomelanin coating in the case of growth in MMT and MMT + pyroquilon.

When *A. alternata* was cultured in MMP, with or without pyroquilon, we observed numerous holes in the cell wall (Figure 6C). These were similar in appearance to those observed in MM + pyroquilon (Figure 6D) although in this condition were less frequent. We interpret these results as an indication of a damaged cell wall in the absence or reduced amounts of DHN-melanin.

**FIGURE 7:**
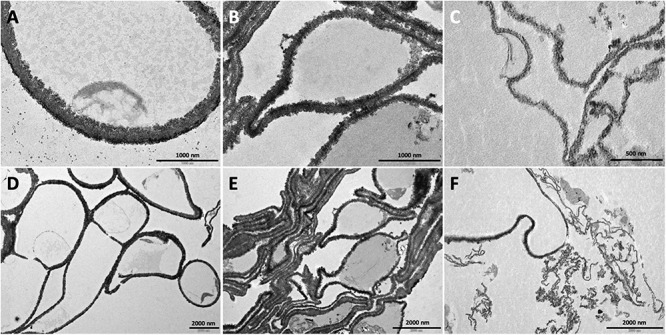
Transmission electron micrograph of the melanin ghosts prepared from *A. alternata* hyphae grown in MM **(A,D)**, MMT **(B,E)** and MMP **(C,F)**. Bars, 1,000 nm in **(A–C)** and 2,000 nm in **(D–F)**.

Melanin particles of hollow spherical shape can be isolated from melanized cells by digestion in concentrated acid and are called “ghosts” because they retain the shape and dimensions of the parent cell due to the melanin structure, as previously performed in the yeast *Cryptococcus neoformans* (Rosas et al., 2000; Dadachova et al., 2007). Although *A. alternata* is a filamentous fungus, we performed this procedure and obtained melanin ghosts from fungus grown in MM, MMT and MMP. The resulting structures from *A. alternata* grown in MM did not fully retain hyphal shape but did retained the septa in a cell wall structure that was more spherical (Figure 7D). In contrast, when *A. alternata* was grown in MMT or MMP, no septa were observed, the cell walls collapsed and the remaining structures aggregated (Figures 7E,F). Growth in MM also resulted in the deposition of melanin granules along the cell wall according to a gradient, with the density of these particles increasing from the inner to the outer layers of the cell wall (Figure 7A). The L-tyrosine (MMT) or L-phenylalanine (MMP) metabolism led to alterations of this pattern, such that the density of melanin granules increased in the center of the electron-dense layer and then decreased along the inner and outer layers (Figures 7B,C, respectively).

### Effects of L-Tyrosine and L-Phenylalanine Metabolism on Cell Wall Chitin and β-Glucan Content

**FIGURE 8:**
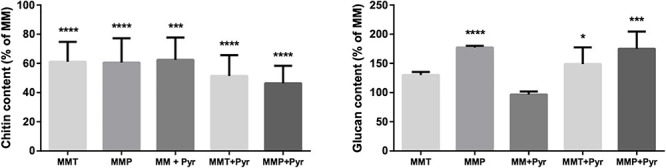
Cell wall components levels in *A. alternata* grown in the presence and absence of L-tyrosine or L-phenylalanine metabolism. Chitin and β-1,3-glucan contents of *A. alternata* are shown. *A. alternata* was cultivated in liquid cultures for 3 days with alternating 8-h light and 16-h dark periods. Results are the mean ± standard errors of the means (SEMs) of triplicates of three independent experiments (Analysis of variance was performed by One-way ANOVA and followed by Tukey multiple comparison test, Prism): *P < 0.05; ***P < 0.001; ****P < 0.0001.

Since growth in MMT and MMP impairs the cell wall DHN-melanin content (Figure 3), we aimed to appraise the modulation of other cell wall components. For that, the cell wall chitin and β-glucan contents were quantified in *A. alternata* mycelia obtained from MM, MMT-, and MMP-grown fungus. The absence of DHN-melanin synthesis (MM + Pyr) inhibited the chitin content to 62.5 ± 6.8% when *A. alternata* is grown in minimal media (MM; control conditions). The chitin content in the cell walls of fungi grown in MMT and MMP was similar, but corresponding to half of that quantified under control conditions (MM) (Figure 8). Adding pyroquilon to MMP and to MMT only decreased slightly the amount of chitin (not statistically significant). The glucan content followed the opposite pattern (Figure 8); in fact, MMP- and MMT-grown fungi had more glucan than in the MM-grown condition (130.2 ± 5.2 and 177.2 ± 2.9%, respectively) and adding pyroquilon did not significantly altered cell wall glucan content in any condition tested (Figure 8).

## Discussion

DHN-melanin is recognized by MelLec receptors in the human host (Stappers et al., 2018) and melanins are considered virulence factors since this pigment protects fungal cells against the attack by immune effector cells by diverse mechanisms, such as inhibiting cytokine production in the host or by scavenging free radicals (Chai et al., 2010; Eisenman and Casadevall, 2012; Heinekamp et al., 2013). Furthermore, melanin makes the fungal cell wall stronger, thus conferring protection against antifungals (Fernandes et al., 2015). The anchorage of melanin to the cell wall has been associated to the cell wall chitin content and structure, another component that is responsible for the cell wall strength (Lenardon et al., 2010). In fact, in both *Candida albicans* and *Cryptococcus neoformans* the supplementation with GlcNAc, which results in increased synthesis of cell wall chitin, impacts melanisation (Walker et al., 2010; Camacho et al., 2017). In this context, studies on the process of melanisation and on the inhibition of melanin synthesis in fungi are of particular importance for the elucidation of pathogenesis mechanisms and identification of new drug targets.

**FIGURE 9:**
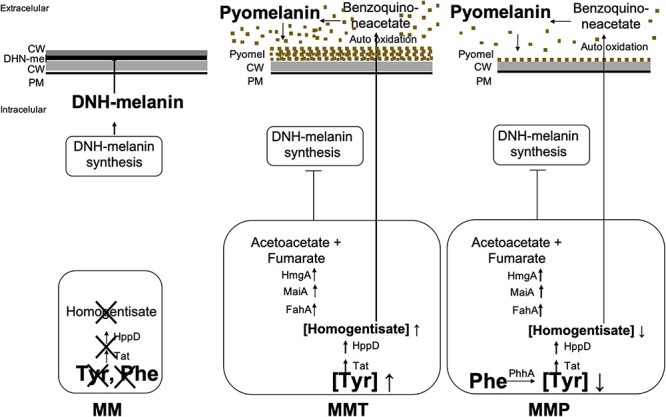
Regulation of pyomelanin synthesis in *A. alternata*. Here, we report that when *A. alternata* is grown in MM medium, no precursor of HGA is present, thus there is no formation of pyomelanin and the DHN-melanin pigment is formed. In MMT medium, L-tyrosine leads the production of large concentrations of HGA that accumulates in the cell and is released into the medium leading to the formation of pyomelanin. There is inhibition of DHN-melanin synthesis. The fungus turns brown. In MMP medium, L-phenylalanine, a L-tyrosine precursor is progressively converted into L-tyrosine by PhhA and also leads to the formation of HGA, but to a lower extent than with L-tyrosine supplemented into the medium. This is probably due to the progressive conversion of L-phenylalanine into L-tyrosine. As L-tyrosine is formed, it is converted into hydroxyphenylpyruvate that is then converted into HGA. This compound is degradated into fumarate and acetoacetate (catabolism of L-tyrosine pathway) leading, consequently to low HGA accumulation and efflux out of the cell and low pyomelanin formation. The DHN-melanin synthesis is inhibited leading to an albino-like phenotype.

In the present work, we report for the first time that *A. alternata* produce another melanin-type pigment in addition to DHN-melanin, which was identified as pyomelanin, using Fourier transform infrared spectroscopy (FTIR). This pigment derives from L-tyrosine through formation of 4-hydroxyphenylpyruvate and HGA (Figure 3; Fernández-Cañón and Peñalva, 1995; Schmaler-Ripcke et al., 2009). Depending on the HmgA activity, HGA accumulates and is secreted from the cell (Yabuuchi and Ohyama, 1972) and then, after auto-oxidation and self-polymerization, forms a brown pigment, pyomelanin (Rodríguez et al., 2000). Here we establish that in *A. alternata* this pigment is produced from the beginning of culture growth and is released into the culture medium, and deposited on the hyphal surface as observed by SEM and TEM, leading to dark brown hypha. The addition of sulcotrione, a HppD inhibitor, to *A. alternata* culture medium with L-tyrosine reduced pyomelanin formation but did not abolished it totally, questioning the specificity of this inhibitor for the HppD from *A. alternata*. L-phenylalanine, a L-tyrosine precursor also induced the formation of pyomelanin, but to a lower extent than L-tyrosine, leading to an albino-like phenotype. This is probably due to the progressive conversion of L-phenylalanine into L-tyrosine. As L-tyrosine is formed, it is converted into hydroxyphenylpyruvate that is then converted into HGA. This compound is broken down into fumarate and acetoacetate (catabolism of L-tyrosine pathway) leading, consequently to low HGA accumulation and low pyomelanin formation (Figure 9). This effect seems independent of amino acid catabolism in general, since neither L-valine and L-glutamine changed the melanisation pattern.

Pyroquilon has the same mechanism of action as other DHN-melanin synthesis inhibitors like tricyclazole, since it inhibits the hydroxynaphtalene reductases, which leads to the accumulation of the shunt products flaviolin and 2-hydroxyjuglone (Wheeler and Klich, 1995). When *A. alternata* was grown in MM medium with pyroquilon, the hyphae were not black and we observed the release and accumulation into the medium of carmine red compounds. Earlier we described this effect of pyroquilon in *A. infectoria*, which was also reported in other fungi, providing evidence that the DHN-melanin biosynthesis is inhibited (Geis et al., 1984; Fernandes et al., 2015). However, when *A. alternata* was grown in minimal media supplemented with L-tyrosine (MMT) with pyroquilon or with L-phenylalanine (MMP) with pyroquilon, we did not observe the generation of these products, which lead us to conclude that DHN-melanin was not synthesized by *A. alternata* during L-tyrosine or L-phenylalanine metabolism. Moreover, under these conditions, we observed a significative decrease in the expression of the genes encoding enzymes of the DHN-melanin pathway. In fact, two genes, encoding a polyketide synthase (*PKSA*) and a 1,3,8-trihydroxynaphthalene reductase (*BRM2*), along with their transcription factor, CmrA, that compose a small gene cluster were down-regulated as well as a scytalone dehydratase encoding gene (*BRM1*), located elsewhere in the genome but that is also under the control of this transcription factor, CmrA (Fetzner et al., 2014).

In addition to the synthesis of pyomelanin under L-tyrosine and L-phenylalanine metabolism, we also report modifications of *A. alternata* cell wall structure and surface decorations depending on the synthesis of the different types of melanin. In recent years, the importance of the cell wall surface in the interaction of filamentous fungi with the host has become a subject of great interest together with surface differences among fungal species (Aimanianda et al., 2009; Rambach et al., 2015; Gow et al., 2017). Most of the current knowledge on melanin interactions with host receptors was obtained with *A. fumigatus*, where the conidia are covered with a proteinaceous layer of hydrophobins and hyphae are covered with polysaccharides (Aimanianda et al., 2009; Rambach et al., 2015). The information regarding the cell wall organization of fungi belonging to the genus *Alternaria* is scarce. Here, we noted extensive surface alterations in *A. alternata* hypha using SEM and TEM when the fungus was grown under the tested conditions and in the presence of a DHN-melanin synthesis inhibitor. In MMT, *A. alternata* was coated with a superficial electron-dense layer, probably made up with pyomelanin, that did not vanish even in the presence of pyroquilon, indicating that this layer is not DHN-melanin. In MMP, in the presence or absence of pyroquilon, the fungal cell walls present a smooth surface with high number of holes. In fact, under low melanotic pigment conditions (MM + pyr, MMP and MMP + Pyr) the fungi exhibit holes in the cell wall structure. Based on this observation there is a high likelihood that DHN-melanin or pyomelanin play a role in *A. alternata* hyphal cell wall final assembly and robustness. Consistent with this notion, when *A. alternata* was grown in MM + pyroquilon, the hyphal cell walls were smoother, thinner, and lighter than when melanin was synthetized. We also prepared melanin ghosts from *A. alternata* hyphae previously grown in MM, MMT or MMP. In contrast to the ghosts produced from the fungi grown in MM, the melanin ghosts from the *A. alternata* cultured in MMT and MMP collapsed and aggregated, indicating that DHN-melanin was required for cell wall strength and proper architecture. Although there are strong indications that melanin is essential for cell wall organization and function (Fernandes et al., 2015; Gow et al., 2017), here we show how depending on the melanin synthetized, the cell wall is modulated. Moreover, we observed that the cell wall chitin decreased significantly under pyomelanin synthesis, when no DNH-melanin was synthetized, and that this decrease was compensated by increasing β-glucan levels, possibly as a salvage compensatory mechanism. Otherwise, under MM growth, the higher chitin contents associated to DHN-melanin biosynthesis can be important as a scaffold to cross-link DHN-melanin to the cell wall components, as demonstrated previously in other fungi that instead of DHN-melanin synthetize L-DOPA-melanin (Walker et al., 2010; Camacho et al., 2017). Consistent with this, for both *Candida albicans* and *Cryptococcus neoformans*, supplementation with GlcNAc increases the synthesis of the cell wall chitin, and affects melanisation (Walker et al., 2010; Camacho et al., 2017). Similarly, it has been demonstrated that the composition of the cell wall and its molecular arrangement are critical factors for the anchoring of melanin in *C. neoformans* and *C. gattii* (Banks et al., 2005; Chrissian et al., 2020). Putting together those observations with our results in this study suggests that the association between melanin and chitin synthesis may be a general principle in the fungal kingdom. Yet, during specific environmental conditions, such as L-tyrosine and L-phenylalanine metabolism, when DHN-melanin is not produced, chitin may not be as necessary to provide a cell wall structure acting as a scaffold for this pigment. In growth conditions that bolster pyomelanin synthesis, we anticipate that this soluble pigment does not require anchorage; it is released into the external medium and then polymerizes to the cell wall. Nevertheless, under these conditions, a lower chitin turns the fungal cell wall more fragile, as attested by SEM and TEM.

In summary, we report that changing growth conditions and the availability of the amino acids L-tyrosine and L-phenylalanine results in profound changes to *A. alternata* pigment production and cell wall architecture (summarized in Figure 9). Our major finds were that: (1) DHN-melanin production is inhibited during pyomelanin synthesis; (2) the absence of DHN-melanin accumulation is associated to a significant reduction in cell wall chitin content; and (3) that the cell wall architecture is affected by which type of melanin is being synthetized. Given that *A. alternata* is an opportunistic agent of animal/human infection that is almost certain to encounter, during infection, nutritional environments where the amino acids L-tyrosine and L-phenylalanine are available, there is a high likelihood that similar pigment responses occur *in vivo*. These changes could influence the cell wall structure, and thus impact the robustness and preparedness of the fungal cell when it faces host defenses, which in turn could affect the outcome of colonization/infection.

## Data Availability Statement

The original contributions presented in the study are included in the article/supplementary material, further inquiries can be directed to the corresponding author/s.

## Author Contributions

CF performed the design of the work, all the experimental procedures (except HPLC and electron microscopy acquisition), analysis, interpretation of data, and most of the writing and revision of the manuscript. MM contributed for the gene expression quantification. LB, MD, and IF performed HPLC analysis. AP carried out Scanning Electron Microscopy. AC participated in the writing and critically revised the work highlighting the scientific impact of the work. TG contributed to the conception and design of the work, funding, analysis and interpretation of data, and writing and revision of the manuscript. All authors contributed to the article and approved the submitted version.

## Conflict of Interest

The authors declare that the research was conducted in the absence of any commercial or financial relationships that could be construed as a potential conflict of interest.

## Publisher’s Note

All claims expressed in this article are solely those of the authors and do not necessarily represent those of their affiliated organizations, or those of the publisher, the editors and the reviewers. Any product that may be evaluated in this article, or claim that may be made by its manufacturer, is not guaranteed or endorsed by the publisher.

## Footnotes

1
http://www.fgsc.net/aspergillus/protocols/mediaforaspergillus.pdf

2
http://genome.jgi.doe.gov

## Abbreviations

MM: minimal salt medium
MMT: minimal salt medium supplemented with L-tyrosine
MMP: minimal salt medium supplemented with L-phenylalanine.
